# Barriers to completing the 4AT for delirium and its clinical implementation in two hospitals: a mixed-methods study

**DOI:** 10.1007/s41999-021-00582-5

**Published:** 2021-11-16

**Authors:** Abdullah A. O. Alhaidari, Kyriakos P. Matsis

**Affiliations:** 1 Department of Geriatrics, Capital and Coast District Health Board, Wellington, New Zealand; 2 Department of Surgery and Anaesthesia, University of Otago, Wellington, New Zealand; 3 Department of Medicine, Capital and Coast District Health Board, Wellington, New Zealand

**Keywords:** Implementation science, Guideline adherence, Delirium, Hospitals, New Zealand

## Abstract

**Purpose:**

To assess the clinical implementation and barriers to completing the 4AT for delirium in general medical and geriatric patients over 75 years upon admission to Wellington and Kenepuru Hospitals during the first eight months of 2017, 2018 and 2019.

**Methods:**

Retrospective data from electronic health records were analysed using an explanatory-sequential mixed-methods approach. The initial quantitative phase measured doctors’ adherence to the 4AT and the rate of positive 4ATs (≥ 4). The subsequent qualitative phase identified doctors’ main reasons for omitting the 4AT through conventional content analysis.

**Results:**

The quantitative population included 7799 acute admissions (mean age 84, 58.2% female). There was good clinical implementation of the 4AT, evidenced by an overall adherence rate of 83.2% and a rate of positive 4ATs of 14.8% that is in keeping with expected delirium rates in similar settings. The qualitative sample consisted of 875 acute admissions (mean age 84, 56.3% female) with documented reasons for omitting the 4AT. The main barriers to completing the 4AT were: reduced patient alertness, communication barriers (language, deafness, aphasia and dysarthria), prioritising patients’ wellness and comfort (addressing critical illnesses, symptoms, end-of-life issues and promoting sleep), pre-existing cognitive disorders, and unstructured delirium assessments.

**Conclusion:**

Adherence to the 4AT was high and sustainable in both hospitals. Most barriers to completing the 4AT were potentially avoidable. Education about the 4AT in relation to these barriers may improve its implementation.

## Key summary points

**Aim:**

To assess the clinical implementation and barriers to completing the 4AT for delirium in general medical and geriatric patients over 75 years upon admission to Wellington and Kenepuru Hospitals.

**Findings:**

The 4AT is a feasible and sustainable tool for the assessment of delirium in the hospital setting. Most of the identified barriers to completing the 4AT are potentially reversible.

**Message:**

Implementation of the 4AT may improve through education about these barriers and emphasising its validity in specific groups.

## Introduction

Delirium is a neurocognitive disorder characterised by rapid cognitive impairment and inattention caused by a medical condition, drug, toxin, or a combination of insults [1]. Delirium is a common condition that affects up to 64% of inpatients in general medical and geriatric wards [2]. Detecting patients at risk of delirium is important, as they may benefit from preventative interventions [3]. Of equal importance is detecting patients with delirium who require prompt assessment and management of the underlying cause.

Delirium is usually associated with bad outcomes, including dementia, prolonged hospitalisation, institutionalisation, and death [4, 5]. Missing delirium is associated with even worse outcomes with up to a three-fold increase in mortality [6]. Unfortunately, as many as 66% of patients with delirium are missed due to several factors [7]. Delirium is an elusive condition with numerous precipitants, different presentations, and a fluctuating course. Diagnosing delirium requires knowledge, skills, and confidence which are not commonly present among health professionals [8]. Delirium occurs more frequently among older, cognitively, or visually impaired patients who are harder to assess [9].

Due to the significant impact of delirium, guidelines recommend assessing patients at risk with validated tools upon admission [10]. Unfortunately, adherence to these tools and their reliability are severely affected by the lack of training and improper implementation [9]. More hospitals are now using the 4AT (https://www.the4at.com), a tool designed to bypass common barriers to delirium assessment [11]. The 4AT is quick and easy to use, does not require special training, and is validated in several languages and various groups, including patients with drowsiness, dementia, and stroke [12]. The 4AT has become the most commonly used assessment tool for delirium within the United Kingdom, with reported adherence rates as high as 95% [13, 14]. However, there is insufficient knowledge about the clinical implementation of the 4AT, and there are no studies focussing on the barriers to completing this test [12]. Addressing these gaps may improve adherence, detection of delirium, and patient outcomes.

This retrospective study aimed to assess the implementation and identify barriers to completing the 4AT when admitting older adults into general medical and geriatric services in two hospitals in New Zealand (NZ). We used an explanatory-sequential, mixed-methods approach to better understand the context and the research phenomenon [15]. The initial quantitative phase assessed key implementation parameters: (a) doctors’ adherence to the 4AT and (b) rate of positive 4ATs (≥ 4) compared to expected rates of delirium in similar settings. The subsequent qualitative phase focussed on identifying doctors’ main reasons for omitting the 4AT.

## Methods

### Background

In 2013, Capital and Coast District Health Board (CCDHB) implemented a system-wide delirium programme in its two hospitals: Wellington Regional Hospital (WRH) and Kenepuru Hospital (KH). The programme incorporated the Confusion Assessment Method (CAM) diagnostic algorithm to identify patients with delirium [16]. The CAM algorithm is based on four items: (1) acute change or fluctuation; (2) inattention; (3) disorganised thinking; and (4) altered level of consciousness. The CAM scores positive for delirium in the presence of items one and two and either three or four. As per the original study, a positive CAM is 94–100% sensitive and 90–95% specific for delirium when completed by a trained individual based on observations made during an interview that includes a formal cognitive assessment.

As detailed in a previous publication, an evaluation in 2015 raised concerns about CCDHB's delirium programme [17]. Nurses completed the CAM without formal cognitive assessments, which has been shown in a previous study to drop its sensitivity to 19% [9]. Furthermore, nurses did not receive CAM-specific training; they found the CAM challenging and did not believe it influenced their patients' care. In addition, doctors were almost entirely unaware of the delirium programme.

A multidisciplinary team was formed to address these deficits. The team considered improving the implementation of the CAM by adding a cognitive assessment tool and CAM-specific training but realised this might complicate the process and require extensive resources. They searched for an alternative tool and decided to implement the 4AT because it has built-in cognitive tests and does not require special training. The 4AT is based on four items: (1) alertness; (2) AMT4; (3) attention; and (4) acute change or fluctuating course. Completing the 4AT generates a numerical score that classifies patients into three categories: (0) delirium or severe cognitive impairment unlikely; (1–3) possible cognitive impairment; and (≥ 4) possible delirium ± cognitive impairment. In a recent meta-analysis, the 4AT had a pooled sensitivity and a pooled specificity of 88% for the detection of delirium [12].

By December 2016, the 4AT was integrated into doctors’ electronic admission forms as a mandatory assessment for patients ≥ 75 years of age. To finalise these forms, doctors had to specify their patients’ 4AT score (0, 1–3, ≥ 4); omit the 4AT due to (Age < 75); or document a reason for omitting the 4AT in a free text-box (Not done: Other reason, please specify: […]).

There were multiple potential benefits for implementing the 4AT on admission. Firstly, it provides a baseline assessment so that acute cognitive decline is easier to detect. Secondly, it helps to identify patients with cognitive impairment who may benefit from delirium-preventative interventions. Lastly, it identifies patients with delirium who may benefit from prompt assessments and treatment of the underlying cause. The implementation phase involved recruiting clinical champions, providing doctors and nurses with laminated 4AT cards and education on the effects of completing the 4AT on patients’ care, as described in the previous publication [17].

### Settings

This retrospective study included admissions under the general medical service at WRH and the geriatric service at KH. Both hospitals are operated by CCDHB, which according to the 2018 census, served 303,987 people from over 30 ethnicities, most of whom were NZ European (66%), NZ Māori (11.6%), and Samoan (5.4%) [18]. NZ Māori are the indigenous people of NZ, about 95% of whom speak English.

WRH is located in Wellington, the capital city of NZ. It is a major tertiary teaching hospital with > 26 specialities and 440 beds. Seventy beds are allocated to general medicine, whose patient census ranges from 50 to 90 throughout the year. Most of these patients are admitted by medical doctors via the emergency department and the medical admission unit.

KH is located in Porirua city, 24 Kilometres north of WRH. KH has two geriatric wards that provide subacute care to 40 patients outside winter and 48 during winter. Most patients are investigated and stabilised in WRH before their transfer via ambulance to KH, which has limited or no access to specific laboratory and radiological services, particularly after hours. At KH, patients are admitted by House Surgeons and Registrars. These doctors rotate every three to six months between specialities within both hospitals.

### The quantitative phase

Our quantitative population included all acute admissions under general medical and geriatric services for patients ≥ 75 years of age, occurring during the first eight months of 2017, 2018 and 2019. The retrospective data analysis began in September 2019, leaving the last four months of 2019 without data. In order for the data between each year to not be affected by seasonal variation in admissions and changes in doctors’ seniority across each year, the last four months of 2017 and 2018 were not included. There were no exclusion criteria otherwise.

For each admission, we obtained data from Electronic Health Records. We collected patients’ age, sex, ethnicity, the 4AT score if completed, and the documented reason for omitting the 4AT if it was not completed. We processed data using Microsoft Excel. We used descriptive statistics (mean, standard deviation, range) to describe admitted patients’ demographics and doctors’ adherence to the 4AT, noting that admissions may have occurred repeatedly for the same patient. Inferential statistics were not needed, as all acute admissions were included. Ethnicity was classified according to NZ’s Level 2 Ethnic Groups classification protocol [19].

### The qualitative phase

Of the quantitative population, the qualitative sample included all admissions where doctors documented a meaningful reason for omitting the 4AT. This sample was cleansed of admissions where doctors documented a meaningless reason for omitting the test (random numbers, symbols or punctuation marks) or erroneously chose (Age < 75) to bypass the test.

For the qualitative sample, doctors’ documented reasons for omitting the 4AT were analysed using the conventional content analysis method [20]. In this method, codes are induced from the text itself, which is useful in the absence of information about the topic that prevents the development of priori codes. For the first analysis endeavour, a geriatrician who lead the development of the delirium programme (first author) and a medical registrar separately analysed doctors’ responses from WRH. All responses were read as a whole to achieve immersion. Responses were then read word by word while highlighting key concepts, such as “drowsy”, “unconscious”, “obtunded”, “agitated”. Similar concepts were then labelled with a representative code, such as “altered mental state”. Following this, both doctors compared their findings and agreed on an initial coding scheme.

Further analysis was solely conducted by the geriatrician. Doctors’ responses from both hospitals were re-analysed. Responses were read word by word, and codes were manually assigned. This process was repeated as new concepts were identified and new codes were defined. For instance, the code “altered mental state” was replaced by either “hyperactive” or “reduced alertness”. Similar codes were then grouped into categories. In search of inferences and connections, codes and categories were examined on screen, on paper, as a whole, according to site (WRH vs KH) and according to time (2017 vs 2018 vs 2019). As new connections were found, categories were split, merged or redefined. This process was reiterated through periods of immersion and distancing until reasonable categories (explanations) for omitting the 4AT were reached. Throughout this process, a codebook was maintained to prevent coders drift (see Online Resource 1: Codebook). In the end, categories, codes and representative quotes were presented to a select group of doctors from CCDHB, who suggested some changes, then validated the interpretation of data in two separate meetings (see acknowledgements).

Descriptive statistics were used to describe admitted patients’ and respondent demographics, noting that admissions may have occurred repeatedly for each patient. Doctors were classified according to position as Trainee Interns, House Surgeons, Registrars and Senior Medical Officers. During the study period, a doctor whose position changed was treated as a unique individual. However, a doctor who worked in the same position in both hospitals was not counted more than once when presenting data for both hospitals. In addition, the number of doctors contributing to each category was counted to indicate its magnitude. However, a doctor who contributed multiple times to the same category was not counted more than once.

The Hutt Valley and Capital and Coast District Health Boards Research Office approved this study and did not require formal ethical review. The authors report no financial conflicts of interest and have not received funding from any agency.

## Results

The quantitative population consisted of 7799 admissions; their mean age was 84 years (standard deviation 5.9, range 75–109), 58.2% (4540) were female, and 62.8% (4899) were NZ European. The quantitative populations’ demographics and ethnic breakdown are demonstrated in Table 1 and Online Resource 2, respectively.

**Table 1:** Population demographics for the quantitative study

Admissions, *n* (%)	Wellington hospital 6217 (79.7%)		Kenepuru hospital 1582 (20.3%)		Both hospitals 7799 (100%)	
Age on admission (years)						
Mean age ± SD (Range)	84 ± 5.9 (75–109)		85 ± 5.8 (75–108)		84 ± 5.9 (75–109)	
Sex, *n* (%)						
Female	3568	(57.4%)	972	(61.4%)	4540	(58.2%)
Male	2649	(42.6%)	610	(38.6%)	3259	(41.8%)
Ethnicity, *n* (%)						
New Zealand European	3842	(61.8%)	1057	(66.8%)	4899	(62.8%)
Other European	1226	(19.7%)	302	(19.1%)	1528	(19.6%)
New Zealand Maori	209	(3.4%)	45	(2.8%)	254	(3.3%)
Other	940	(15.1%)	178	(11.3%)	1118	(14.3%)

*SD* Standard deviation

Of the 7799 admissions; 83.2% (6492) had a 4AT completed, 11.2% (875) had a meaningful reason for omitting the 4AT, and 5.5% (432) did not have a meaningful reason for omitting it. The latter group included 42 admissions to WRH where doctors bypassed the test by erroneously choosing (Age < 75). Of the 7799 admissions; 14.8% (1154) had a positive 4AT score (≥ 4). Doctors’ adherence to the 4AT and the 4AT scores are demonstrated in Table 2 and Online Resource 3, respectively.

**Table 2:** Doctors’ adherence to the 4AT

4ATs completed, *n* (%)	2017^a^		2018^a^		2019^a^			Total	
Wellington Hospital	1662	(79.8%)	1618	(82.9%)	1796		(82.3%)	5076	(81.6%)
Kenepuru Hospital	516	(89.1%)	476	(88.3%)	424		(91.4%)	1416	(89.5%)
Both Hospitals	2178	(81.8%)	2094	(84.1%)	2220		(83.9%)	6492	(83.2%)

^a^Figures represent 4ATs completed during the first eight months of the years 2017, 2018 and 2019.

The qualitative sample consisted of 875 admissions; their mean age was 84 years (standard deviation 5.9, range 75–108), 56.3% (493) were female, and 44.9% (393) were NZ European. The qualitative samples’ demographics and ethnic breakdown are demonstrated in Table 3 and Online Resource 4, respectively.

**Table 3:** Sample demographics for the qualitative study

Admissions, *n* (%)	Wellington hospital 757 (86.5%)		Kenepuru hospital 118 (13.5%)		Both hospitals 875 (100%)	
Age on admission (years)						
Mean age ± SD (Range)	84 ± 5.8 (75–102)		84 ± 6.2 (75–108)		84 ± 5.9 (75–108)	
Sex, *n* (%)						
Female	424	(56%)	69	(58.5%)	493	(56.3%)
Male	333	(44%)	49	(41.5%)	382	(43.7%)
Ethnicity, *n* (%)						
New Zealand European	337	(44.5%)	56	(47.5%)	393	(44.9%)
Other European	132	(17.4%)	22	(18.6%)	154	(17.6%)
New Zealand Maori	24	(3.2%)	6	(5.1%)	30	(3.4%)
Other	264	(34.9%)	34	(28.8%)	298	(34.1%)

*SD* Standard deviation

The qualitative sample was generated by 211 doctors; 63% (132) were Registrars, 32% (68) were House Surgeons, 3% (6) were Senior Medical Officers, 1% (2) were Trainee Interns, and 1% (3) could not be identified. Of these respondents, 52% (109) were female, 42% (89) were male, and 6% (13) could not be identified. Thirty-three doctors worked in the same position in both hospitals. Nine doctors worked as a House Surgeon then as a Registrar. Doctors’ demographics are demonstrated in Table 4.

**Table 4:** Responding doctors demographics

Doctors, *n*	Wellington Hospital		Kenepuru Hospital		Both hospitals^a^	
	172		72		211	
Position, *n* (%)						
Registrar	116	(67%)	37	(51%)	132	(63%)
House Surgeon	47	(27%)	33	(46%)	68	(32%)
Senior Medical Officer	5	(3%)	1	(1%)	6	(3%)
Trainee Intern	2	(1%)	0	(0%)	2	(1%)
Unknown	2	(1%)	1	(1%)	3	(1%)
Sex, *n* (%)						
Female	91	(53%)	34	(47%)	109	(52%)
Male	75	(44%)	30	(42%)	89	(42%)
Unknown	6	(3%)	8	(11%)	13	(6%)

^a^Each of the 33 doctors who worked in the same position in both hospitals was not counted more than once

By analysing doctors’ 875 responses, five main categories emerged: (1) reduced patient alertness; (2) communication barriers; (3) prioritising patients’ wellness and comfort; (4) pre-existing cognitive disorders; and (5) unstructured delirium assessments. The main categories, subcategories and representative quotes are demonstrated in Table 5.

1. *Reduced patient alertness (51 doctors)*: In this category, doctors omitted the 4AT due to reduced patient alertness. Doctors reported this for varying degrees of severity, including the “drowsy”, “obtunded”, “unresponsive”, “comatose”, and those with reduced Glasgow Coma Scale scores [21]. Doctors reported it was impossible to test these patients, as they were “not conscious enough” or “too drowsy to assess”.
2. *Communication barriers (127 doctors)*: In this category, doctors omitted the 4AT due to factors other than reduced patient alertness that prevented the exchange of information with their patients.
  One hundred and twelve doctors cited language as a barrier in this category. Some doctors felt it was difficult, or even impossible, to complete the test in patients with Limited English. Some blamed themselves for not speaking their patients’ language, and others blamed a lack of interpreters. Nevertheless, language remained a barrier, even in the presence of family interpreters who were relied on during the consultation process. One Registrar feared they might disadvantage their patient if the test was completed given their limited English. This language barrier was mainly accounted for by Indians (20%), Chinese (19%), Samoans (17%), Non-NZ Europeans (13%), Southeast Asians (7%) and Middle Easterners (7%). NZ Māori did not contribute at all to this subcategory.
  Thirty-two doctors found it difficult to test patients with aphasia and dysarthria, mainly in the context of stroke or dementia. Eleven doctors did not test patients due to deafness. Twenty-four doctors did not test patients due to non-specific communication difficulties.
3. *Pre-existing cognitive disorders (61 doctors)*: In this category, the mere knowledge of a pre-existing cognitive disorder was sufficient for doctors to omit the 4AT. This was reported in varying degrees of severity, from mild cognitive impairment to advanced dementia, and in various types of dementia, including Alzheimer’s and Lewy body.
4. *Unstructured delirium assessments (48 doctors)*: In this category, doctors relied on their general observations of the patient and clinical gestalt instead of a standard assessment tool to diagnose or rule out delirium. Clinical gestalt is where doctors make a clinical decision based on their previous knowledge and experience with a condition in the absence of complete information about their patient. Some doctors relied on the presence or absence of cardinal features for delirium, such as being alert or the lack of acute change. Some relied on their patients’ ability or inability to provide a clear history. Another group of doctors relied on their own feelings and how the patient appeared to them during the consultation.
5. *Prioritising patients' wellness and comfort (81 doctors)*: In this category, doctors omitted the 4AT to avoid the burden associated with testing or provide urgent and comfort-based interventions to unwell, symptomatic, dying or sleepy patients. Forty-four doctors omitted the test in acutely ill and breathless patients, some of whom required urgent resuscitation or respiratory support. Seventeen doctors avoided testing patients already suffering from physical symptoms or emotional distress, such as fatigue, anxiety, nausea, or pain. Twenty-two doctors avoided testing patients who were terminally ill or dying. They felt it was insensitive to test this group or prioritised discussing advanced care plans and the provision of end-of-life cares over the 4AT. Twenty-one doctors omitted the test due to sleep; some respected their patients’ wishes to sleep, some did not wake their patients, and some woke them up for a brief assessment which did not include the 4AT.

**Table 5:** Main barriers to completing the 4AT with representative quotes

Main categories	Subcategories	Representative quotes
Reduced alertness		Drowsy Reduced GCS due to severe illness Not conscious enough Too drowsy to assess Reduced level of consciousness prevents screen
Communication barriers	Language barrier	Too difficult with language barrier English second language Language barrier, (…) speaks Samoan and I unfortunately do not Unable to converse with patient no translator Poor English daughter translating Mrs (…) English is limited and unfair assessment
	Aphasia and dysarthria	Difficulty answering questions due to dysphasia post stroke Pt (patient) averbal with severe dementia BG (background) Difficult as patient dysarthric
	Deafness	Very deaf Deaf and Mute
	Non-specific	Unable to communicate with patient
Pre-existing cognitive disorders		Known mild cognitive impairment Known Alzheimer’s Established dementia Known advanced dementia Known cognitive impairment. MOCA 15/30 on 17/6/19 Known Dementia and confusion at baseline according to family Cognitive impairment puts patient at risk of delirium in hospital
Unstructured delirium assessments		[Test] not formally done; but [patient] not delirious Appeared appropriate and not delirious Clinically was felt to have cognitive clouding Patient clearly articulates delirium Not clinically delirious Delirium is confirmed clinically No change from previous admission Not confused per family Not done no concerns about cogitation [*sic*] Alert, appropriate, clear history, not delirious
Prioritising patients' wellness and comfort	Critical illness	Critically unwell Unwell in resus (Resuscitation room) Active bleeding undergoing resuscitation Quite SOB (short of breath) In ED resuscitation (room), struggling to talk Too unwell with resp (respiratory) symptoms
	Symptomatic	Tearful and upset Tremulous and anxious Pt too distressed with pain and nausea Currently vomiting, not clinically appropriate Patient too fatigued
	End of life	Terminal illness Possibly dying Palliative care approach Sensitive discussion around end-of-life Just completed discussions around palliative care and resus
	Sleep	(…) was keen to get some sleep Currently asleep. Will attempt later Woken briefly for review

There were minor reasons for omitting the 4AT. Fourteen doctors deemed the 4AT “irrelevant” without elaborating. Eleven doctors used an alternative tool, such as the Abbreviated Mental Test 4, the Abbreviated Mental Test, the Montreal Cognitive Assessment, and Orientation to Time, Place and Person [22–25]. Other reasons included time constraints, forgetting to test, delirium already diagnosed, patient agitation, patient refusal, being transferred to radiology suite, presenting with a primary neurological or psychiatric disorder, and intoxication.

Adherence across sites was lower at WRH than KH (81.6% vs 89.5%), where critical illness, reduced patient alertness and end-of-life matters were barely reported. Adherence across the three periods was comparable (81.8%, 84.1% and 83.9%), and the barriers were similar.

## Discussion

This mixed-methods study assessed the clinical implementation and barriers to completing the 4AT for 7799 admissions in two hospitals over nearly 3 years. The study demonstrated high adherence to the 4AT (83.2%) and a rate of positive 4ATs (14.8%) in keeping with the reported rates of delirium (14–16%) in similar settings [26]. The study also identified barriers to completing the 4AT, which represent new knowledge and opportunities for improvement, as detailed below.

Reduced patient alertness was a surprising barrier, given that the 4AT was designed to enable the assessment of patients with reduced alertness. Reduced alertness is the first item to investigate when administering the 4AT and is given sufficient power to indicate “probable delirium”. Indeed, a systematic review of delirium tools identified the 4AT as an ideal test for hypoactive delirium [27]. The explanation we found was that some doctors falsely believed the test could not be completed in drowsy patients. This is in keeping with the findings of a multicentric survey, where 72% of practitioners believed verbal responsiveness was essential to diagnose delirium, and 31% reported the 4AT was “difficult” or “very difficult” to complete in drowsy non-verbal patients [28]. In that survey, practitioners were twice as likely to use vague terms, similar to our doctors’ ones, instead of “delirium” to describe drowsy patients. That study emphasised the importance of education, prompted the development of a 4AT-specific guide (https://www.the4at.com/guide) and recommended keeping the scoring and guidance components of the 4AT when digitalised.

Language was the most common reason for omitting the 4AT. There is limited research about delirium and the 4AT in patients who do not speak the local language [27]. The only study that focused on this group found the 4AT to be quite sensitive (91%) for delirium in patients from non-English speaking backgrounds in the presence of professional interpreters [29]. Indeed, the 4AT is simple and can indicate “probable delirium” in the presence of drowsiness or acute change without the need to communicate in a shared language. This, and the increasing number of validated translations of the 4AT, are suggestive of a culturally and linguistically friendly tool [12]. However, more information is needed about the accuracy of the 4AT in different ethnicities and languages. For instance, a previous study reported confusion among pilot surveyors when completing the 4AT in languages such as Mandarin, where numbers represent the months of the year [30]. A particular concern was the reliance on family interpreters despite the availability of professional interpreters for patients with limited English and deafness. This is a common phenomenon according to local and international studies [31, 32]. For both groups, utilising professional interpreters can improve access to healthcare, clinical outcomes and satisfaction [33, 34]. Developing cultural competence, knowing when professional interpreters are required and facilitating their use may address this barrier.

Some of our doctors found it difficult to complete the 4AT in patients with aphasia and dysarthria. In our experience, this was compounded by the lack of familiarity with recent research about delirium and its tools. At CCDHB, concerns were raised about the appropriateness of the 4AT and delirium pamphlets on stroke and stroke rehabilitation wards. These concerns were similar to those voiced in a previous survey, where 87% of stroke practitioners were not sure, or did not believe, that delirium tools were appropriate in acute stroke, and many found them particularly challenging to complete in aphasia [35]. We addressed these concerns by emphasising the prevalence of delirium and the validity of the 4AT in acute stroke. Indeed, the 4AT is now one of the most validated tools with great accuracy in this setting, even in patients with mild to moderate aphasia [36, 37]. Conveying this knowledge to health professionals may overcome this barrier while noting that more research is needed in patients with severe aphasia.

The mere presence of a pre-existing cognitive disorder was an unexpected barrier to completing the 4AT. Potential explanations include unawareness that pre-existing cognitive disorders and delirium may co-exist and that the 4AT may help differentiate between them. The 4AT classifies patients into three groups, which is unusual and might not be straightforward to comprehend without training. This barrier may be addressed by improving doctors’ basic knowledge about delirium, the validity of the 4AT in diagnosing delirium superimposed on dementia, and the impact this diagnosis has on a patient’s journey [11, 29, 38].

Unstructured assessments based on general observation and clinician gestalt has been one of the most favoured and extensively evaluated methods in the assessment for delirium [39, 40]. However, this method is prone to errors, and it depends on the health professionals’ knowledge about delirium, which is generally poor [8]. In a scoping review, the sensitivity of gestalt for detecting delirium ranged from 0 to 81% depending on various factors, including whose gestalt was assessed [39]. Despite the known effects of delirium on attention and memory, it is yet unclear whether a patient’s ability to provide a clear history is accurate enough to rule delirium out [41]. Indeed, unstructured delirium assessments have been deemed inappropriate in various settings, even after educational interventions, and are a common reason for being dismissed [40, 42, 43].

Some doctors prioritised life-saving interventions and symptom control over completing the 4AT on admission. Others avoided the burden associated with testing in palliative or sleepy patients. Previous studies have identified similar barriers when assessing adherence to other delirium tools [43, 44]. It may be argued that life-saving interventions and symptom control are expected to take precedence over delirium assessments upon admission. However, severe illness is a significant risk factor for delirium, and delirium itself is a life-threatening condition that needs to be identified promptly. Therefore, routine assessments are recommended, particularly as most delirious patients suffer from the hypoactive form that is harder to detect [9, 45, 46]. Delirium and its complications are commonly associated with intense and long-lasting distress, which can outweigh the burden of completing a brief assessment [47]. Delirium assessments provide an opportunity to educate patients and their families about delirium, noting the importance of family involvement and that pre-emptive education can significantly reduce delirium-related distress [48]. In this study, most patients were assessed for delirium, which was achievable by implementing the 4AT, a simple, quick and acceptable tool that allows testing unwell patients.

Lower clinical acuity may explain why critical illness, reduced patient alertness and end-of-life matters were not significant barriers at KH and may explain the higher adherence rate. At CCDHB, patients present acutely to WRH, are investigated and stabilised before being transferred to KH for post-acute care. Indeed, a significant association between acute admissions and lower adherence to delirium assessments has been noted before [49].

A few studies suggested that 4AT training is necessary and requested exploring health professionals’ training needs [12, 50]. We suppose that increasing doctors’ general knowledge about delirium, the 4AT, and the barriers identified in this study may improve its implementation. A particular example is the barrier “reduced patient alertness”, which is, in fact, a cardinal feature of delirium.

This study has multiple strengths. A better understanding of the research phenomenon was achieved by mixing quantitative and qualitative methods. Generalisability was increased by including all admissions even for patients with reduced alertness, limited English, deafness, aphasia, dementia, and severe or terminal illness. Authors’ preconceived biases were mitigated by inducing codes and categories directly from doctors’ responses instead of relying on priori codes. Interview bias was minimised through retrospective retrieval of doctors’ responses. Recall bias was minimised by relying on data that was documented at the point of care. Immersion, critical thinking and recognition of misspellings were facilitated by manually coding doctors’ responses. Coder drift was prevented by using a codebook. Validation of findings was achieved through member checking.

This study has several limitations. Content analysis cannot establish causality and is limited, at best, to the identification of key concepts within data. Content analysis may disregard the context of the study, but this was mitigated through the mixed-methods approach. Retrospective retrieval of doctors’ responses limited the ability to ask for clarification, especially for ‘meaningless’ entries, which may represent frustration or ignorance with the 4AT that could not be openly disclosed. Finally, our findings may not be applicable beyond general medical and geriatric settings.

To conclude, this study provides new insight into the barriers to completing the 4AT in general medical and geriatric wards. Identifying these barriers can guide the development of educational materials, which may improve the implementation of the 4AT. Further research is needed to assess the effects of education and training on the implementation of the 4AT.

## Data Availability

Due to privacy and ethical concerns, data cannot be disseminated.
